# Endoscopic Treatment of Staple-Line Leaks After Sleeve Gastrectomy in Patients with Obesity: Which One is the Best Option, if Any? A Systematic Review with Meta-Analysis and Meta-regression

**DOI:** 10.1007/s11695-025-08294-6

**Published:** 2025-10-20

**Authors:** Giuseppe Galloro, Mariano Cesare Giglio, Alessia Chini, Rosa Maione, Matteo Pollastro, Rosa Vitale, Antonio Pisani, Mario Musella

**Affiliations:** 1 Department of Clinical Medicine and Surgery, Digestive Surgical Endoscopy Unit, University of Naples Federico II, Naples, Italy; 2 Department of Clinical Medicine and Surgery, Hepato-Pancreato-Biliary Surgery Unit, University of Naples Federico II, Naples, Italy; 3 Gastroenterology II Unit, Gastroenterology Hospital ‘‘Saverio de Bellis’’, Castellana Grotte (Bari), Italy; 4 Department of Clinical Medicine and Surgery, General Endocrine-Metabolic and Obesity Surgery Unit, University of Naples Federico II, Naples, Italy

**Keywords:** Obesity, Metabolic and bariatric endoscopy, Endoscopic treatment, Systematic review, Meta-analysis, Meta-regression, Laparoscopic sleeve gastrectomy (LSG), Staple-line leak (SLL)

## Abstract

Laparoscopic sleeve gastrectomy (LSG) is the most common bariatric surgery, with staple-line leak (SLL) as its most severe complication. Various endoscopic treatments exist, but no universal algorithm is established. The aim of this paper is to estimate the success rates of different endoscopic techniques for SLL management. A search of Medline, Embase, and SCOPUS identified studies on endoscopic treatments. Pooled success rates were calculated using random-effects modeling. Seventy-nine studies (2205 patients) showed an overall success rate of 84.1%. Double-pigtail drainage (90.07%), endoscopic vacuum therapy (90.2%), and septotomy (88.25%) were most effective. Meta-regression indicated significantly higher success with double-pigtail drainage (*p* = 0.035). Endoscopic treatments, especially double-pigtail drainage and vacuum therapy, are highly effective for SLL. Further research is needed to compare techniques and evaluate additional clinical factors.

**Supplementary Information:**

The online version contains supplementary material available at 10.1007/s11695-025-08294-6.

## Introduction

Laparoscopic sleeve gastrectomy (LSG) is currently the most common bariatric procedure worldwide, accounting for 45–75% of primary interventions in the USA, with favorable outcomes in terms of weight loss [1, 2]. The most serious complication of LSG is a staple-line leak (SLL), with an average reported incidence ranging from 0 to 7% [3], although the trend is decreasing [4]. Despite this, the life-threatening nature of this complication demands careful consideration [5, 6]^.^

Several surgical and non-surgical strategies have been proposed for managing SLL after LSG. The American Society for Metabolic and Bariatric and Surgery (ASMBS) recommends conservative, non-surgical treatment as the first-line approach, with endoscopic procedures—including stenting, pigtail drainage, over-the-scope clips (OTSC) or through-the-scope clips (TTSC), vacuum therapy, septotomy, and tissue sealants representing valuable options [4].

Unfortunately, the available evidence remains inconclusive due to the numerical inconsistency (case reports or short case series) and the high heterogeneity of the most published literature data (very often based on retrospective observational studies). As a result, no universally accepted management algorithm has yet been established [7]. The best evidence could be provided by prospective randomized clinical trials, but these are difficult to perform for practical and ethical reasons.

Therefore, we performed a systematic review with meta-analysis of the most recent literature on the endoscopic treatments for SLL after LSG, including tissue sealants, internal drainage, stenting, clipping, vacuum therapy, septotomy, endoscopic suturing, and occluders.

This paper aims to analyze, on the grounds of the literature-based findings, the different techniques of endoscopic management of staple-line leaks after LSG, assessing and comparing their technical characteristics, successful closure rate, and technical and clinical outcomes.

## Material and Methods

The systematic review and the meta-analysis were performed following the Preferred Reporting Items for Systematic Reviews and Meta-Analyses (PRISMA) and Meta-analysis of Observational Studies in Epidemiology (MOOSE) guidelines [8].

### Search Strategy

A systematic search of the Medline, Embase, and SCOPUS databases from inception to January 11, 2024, was conducted using the terms “sleeve,” “gastrectomy,” “complication,” “leak,” “dehiscence,” “fistula,” “endoscopic,” “management,” and “treatment”; related words; and Medical Headings (Supplementary File 1, Search strategy for Medline). The search was limited to articles published in English, and there was no restriction according to the publication status. The reference list of selected studies was screened to identify additional potentially relevant studies.

### Study Selection

To be selected for the meta-analysis, the studies had to meet the following criteria: (1) to include patients who underwent sleeve gastrectomy and subsequently developed a leak from the staple-line; (2) these patients had to undergo an endoscopic treatment of the leak as first- or second-line treatment; (3) to report outcomes of the endoscopic treatment; (4) to include at least five patients, to prevent instability and imprecision in the meta-analytic model. Conference abstracts and non-human studies were excluded.

### Data Extraction and Quality Assessment

Four reviewers (P.M., C.A., M.R., V.R.) extracted data from each selected study regarding the first author, publication year, country of origin, design, number of included patients with a diagnosis of SLL, SLL classification according to the timing of occurrence (acute, early, and chronic), location (esophagogastric junction), type and characteristics of the endoscopic treatment, if the endoscopic treatment was performed as first- or second-line treatment, if the endoscopic treatment was combined or not with additional treatments, and number of successful cases. If the study reported the success rate of different endoscopic approaches, these data were analyzed separately to estimate the efficacy of different interventions. Similarly, whenever possible, the efficacy of the endoscopic treatments was analyzed according to the location and timing of the occurrence of SLL. The quality of each study was evaluated by a modified version of the Newcastle–Ottawa scale for the assessment of the quality of non-randomized studies [9]. By this tool, each study quality was appraised by exploring four domains related to patient selection, outcome measurement tools, outcome assessment, and adjustment for confounders.

### Statistical Analysis

The primary outcome was the overall success rate of the endoscopic treatments. Secondary outcomes were the success rate of each type of endoscopic treatment, and according to leak location, timing of occurrence, and line of treatment, the complication rate of the endoscopic treatment.

The success rate was calculated by dividing the number of patients achieving success with the endoscopic treatment by the total number of patients undergoing the endoscopic treatment. Pooled rates, expressed as a percentage, rounded to one decimal place, with 95% confidence intervals, were computed using a random-effect model according to the DerSimonian and Laird method [10]. The Freeman-Tukey double arcsine transformation of the prevalence was used to incorporate in the pooled analysis also studies with rates equal to 0% [11]. The presence of heterogeneity among the studies was assessed by the Cochrane *Q* test and quantified with the inconsistency index (*I*^2^), with *I*^2^ values of 25, 50, and 75% considered indicative of low, moderate, and high statistical heterogeneity [12]. To further explore and address heterogeneity, subgroup analyses and random-effects meta-regression (restricted maximum likelihood method) were performed to evaluate the impact of treatment type, leak location, timing of occurrence, and line of treatment on treatment success [13]. Influence analysis was performed by leaving each study out in turn and re-computing the summary effect to assess the impact of each study on the overall estimate and heterogeneity. Publication bias was assessed by using Egger’s linear regression method [14]. A *p*-value < 0.05 was considered statistically significant. R version 3.6.1 (2019, The R Foundation for Statistical Computing) was used for statistical analyses.

## Results

### Study Selection and Characteristics

**Fig. 1:**
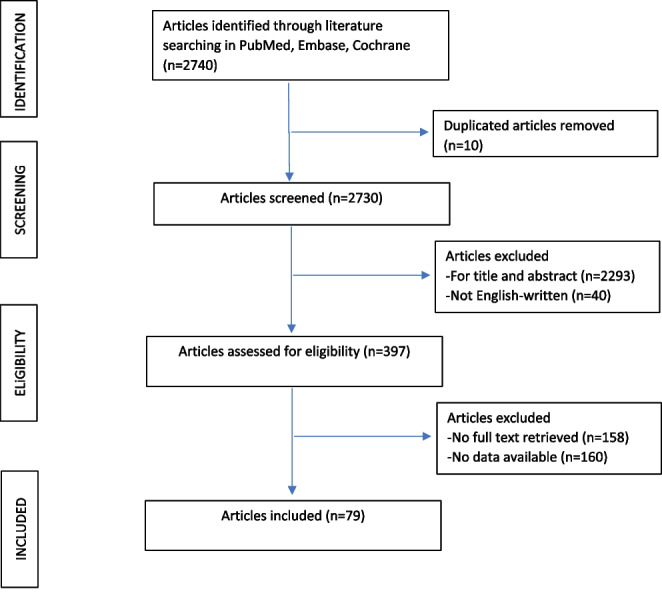
Articles selection flowchart according to preferred reporting items for systematic reviews and meta-analyses (PRISMA)

A total of 79 studies met the inclusion criteria and were selected for this meta-analysis according to Preferred Reporting Items for Systematic Reviews and Meta-analyses (PRISMA) (Fig. 1). The characteristics of the included studies are reported in Table 1. A total of 2205 patients undergoing endoscopic treatment for a staple-line leak following sleeve gastrectomy were included in this meta-analysis. Forty-seven studies reported information regarding the timing of staple-line leak occurrence [2, 7, 15–59], while 48 studies reported information regarding leak location [2, 7, 15–17, 19, 21–31, 34–37, 39, 40, 43–45, 47–52, 54, 55, 57, 58, 60–71].

**Table 1:** Characteristics of the included studies

*STUDY*	Year	Patients (no.)	Country	Treatment type	Details	Success (no.)	Leaks localizations	Time from surgery to leak	Complications (no.)
*Aburajab *et al	2017	5	USA	Stent, Clips	FCSEMS, OTSC	5	-	-	2
*Alamdari *et al	2018	8	Iran	Stent	FCSEMS	7	GOJ = 7; distal = 1	Acute = 1; early = 6; chronic = 1	1
*Assalia *et al	2018	24	Israel	Sealant	Fibrine glue	23	GOJ = 24; distal = 0	Acute = 10; early = 9; chronic = 5	0
*Baretta *et al	2015	9	Brazil	Septotomy	Endoscopic stricturotomy	8	GOJ = 9; distal = 0	-	1
*Archid *et al	2020	8	Germany	EVT	Endoscopic vacuum therapy	7	GOJ = 7; distal = 1	Acute = 0; early = 8; chronic = 0	1
*Manta *et al	2016	6	Italy	Stent, clips	SEMS, OTSC	3	-	Acute = 3; early = 3; chronic = 0	-
*Bona *et al	2020	8	Italy	Stent, clips	Esophageal mega stent, OTSC	7	GOJ = 8; distal = 0	Acute = 0; early = 7; chronic = 1	1
*Bashah *et al	2020	73	Qatar	Stent, clips	Double-pigtail stent, OTSC	66	-	-	2
*Yimcharoen *et al	2011	6	USA	Stent	Fully and partially covered SEMS	4	-	Acute = 2; early = 0; chronic = 4	2
*Aljahdli *et al	2021	11	Saudi Arabia	Stent	FCSEMS	9	GOJ = 10; distal = 1	Acute = 2; early = 8; chronic = 1	5
*Almadi *et al	2018	64	Saudi Arabia	Stent	Fully and partially covered SEMS	60	GOJ = 64; distal = 0	Acute = 32; early = 29; chronic = 3	9
*Boerlage *et al	2018	13	Netherlands	Stent	FCSEMS	9	-	-	3
*Périssé *et al	2015	23	Brazil	Stent	FCSEMS	19	GOJ = 23; distal = 0	-	6
*Alazmi *et al	2014	17	Kuwait	Stent	SEMS, SEPS	13		Acute = 10; early = 6; chronic = 1	6
*Al-Kurd *et al	2018	14	Israel	Stent, clips	OTSC, non-OTSC clip, stent	6	-	-	-
*Billman *et al	2021	23	Germany	Stent	Esophageal mega stent, FCSEMS	16	-	-	7
*Archid *et al	2021	27	Germany	Stent, EVT	SEMS, EVT	17	GOJ = 26; distal = 1	Acute = 7; early = 20; chronic = 0	12
*Balagué *et al	2021	44	Spain	Stent, clips, sealant	SEMS, OTSC, TTS, sealant	28	GOJ = 39; distal = 5	Acute = 18; early = 26; chronic = 0	25
*Caiazzo *et al	2020	100	France	Stent	Double-pigtail stent, covered stent	4	GOJ = 79; distal = 21	Acute = 60; early = 33; chronic = 7	-
*Benosman *et al	2018	26	France	Stent, clips	Double-pigtail stent, SEMS, TTS, OTSC	23	GOJ = 26; distal = 0	-	12
*Balagué *et al	2020	42	Spain	Stent	Fully and partially covered SEMS	23	-	-	18
*Fuentes-Valenzuela *et al	2021	5	Spain	Stent	Double-pigtail stent	5	GOJ = 5; distal = 5	Acute = 3; early = 1; chronic = 1	1
*Garofalo *et al	2017	11	Canada	Stent, clips	Double-pigtail stent, SEMS, OTSC	11	GOJ = 11; distal = 0	Acute = 2; early = 8; chronic = 1	2
*Hany *et al	2021	44	Egypt	Stent	Covered SEMS	33	GOJ = 35; distal = 9	Acute = 0; early = 44; chronic = 0	11
*Diaz *et al	2020	5	USA	Septotomy	Endoscopic stricturotomy	5	GOJ = 5; distal = 0	Acute = 1; early = 4; chronic = 0	-
*Foo *et al	2017	6	Australia	Stent	Double-pigtail stent, FCSEMS	6	GOJ = 6; distal = 0	-	-
*Ferraz *et al	2021	21	Brazil	Stent	SEMS	20	GOJ = 19; distal = 2	Acute = 5; early = 16; chronic = 0	1
*Casella *et al	2009	6	Italy	Stent, sealant	Covered stent, glue	6	GOJ = 5; distal = 1	Acute = 3; early = 3; chronic = 0	0
*Donatelli *et al	2015	67	France	Stent	Double-pigtail stent	59	GOJ = 56; distal = 11	Acute = 26; early = 32; chronic = 9	6
*Guzaiz *et al	2016	12	Saudi Arabia	Stent	Covered SEMS	12	-	-	6
*de Moura *et al	2019	37	Brazil	Stent	Covered SEMS	29	GOJ = 35; distal = 2	Acute = 10; early = 27; chronic = 0	4
*Fishman *et al	2015	26	Israel	Stent	SEMS	17	GOJ = 25; distal = 1	Acute = 1; early = 17; chronic = 8	5
*Donatelli *et al	2014	21	France	Stent	Double-pigtail stent	12	GOJ = 19; distal = 2	-	2
*Donatelli *et al	2021	275	France	Stent	SEMS, double-pigtail stent	246	-	-	-
*Csendes *et al	2010	16	Chile	Endoscopic treatment	-	16	GOJ = 14; distal = 2	Acute = 7; early = 9; chronic = 0	1
*Christophorou *et al	2015	110	France	Stent, clips, sealant	FCSEMS, PCSEMS, double-pigtail stent, OTSC, glue	81	-	Acute = 50; early = 42; chronic = 18	18
*Gonzalez *et al	2015	15	France	Stent	Mega stent	12	-	-	-
*Gonzalez *et al	2018	44	France	Stent	Double-pigtail stent	37	-	Acute = 3; early = 33; chronic = 8	11
*El-Sayes *et al	2017	27	Egypt	Stent	Covered SEMS	23	-	-	-
*Lazzarin *et al	2020	5	Italy	Stent	Double-pigtail stent	5	GOJ = 5; distal = 0	Acute = 3; early = 2; chronic = 0	0
*Mahadev *et al	2017	9	USA	Septotomy	Endoscopic stricturotomy	6	GOJ = 9; distal = 0	-	0
*Mohammad *et al	2019	8	Egypt	Stent	Mega stent	7	GOJ = 5; distal = 3	Acute = 4; early = 4; chronic = 0	1
*Klimczak *et al	2018	14	Poland	Stent	Mega stent	10	GOJ = 14; distal = 0	Acute = 1; early = 12; chronic = 1	9
*Krishnan *et al	2019	16	USA	Stent	SEMS	15	-	-	2
*Talbot *et al	2017	21	Australia	Stent, clips	FCSEMS, PCSEMS, TTS, OTSC	18	-	-	3
*Leenders *et al	2013	6	Netherlands	Stent	FCSEMS	5	GOJ = 3; distal = 3	Acute = 3; early = 3; chronic = 0	2
*Moon *et al	2015	15	USA	Stent, clips, sealant	SEMS, TTS, fibrine glue	14	GOJ = 15; distal = 0	Acute = 3; early = 9; chronic = 3	-
*Lamb *et al	2020	5	USA	Stent, endoscopic suturing	-	5	-	Acute = 0; early = 4; chronic = 1	1
*Pequignot *et al	2012	25	France	Stent	Double-pigtail stent, covered stent	19	GOJ = 19; distal = 6	Acute = 14; early = 11; chronic = 0	9
*Mercky *et al	2015	18	France	Clips	OTSC, non-OTSC clip, stent	18	-	Acute = 0; early = 5; chronic = 13	6
*Leeds *et al	2016	9	USA	EVT	Endoscopic vacuum therapy	8	-	Acute = 1; early = 3; chronic = 4	2
*Keren *et al	2015	26	Israel	Clips	OTSC	21	GOJ = 22; distal = 4	Acute = 10; early = 15; chronic = 1	0
*del Campo *et al	2018	24	USA	Stent	SEMS	14	GOJ = 18; distal = 6	Acute = 5; early = 8; chronic = 9	10
*Olmi *et al	2020	66	Italy	Stent, clips	SEMS, double-pigtail stent, OTSC	63	GOJ = 53; distal = 13	Acute = 30; early = 30; chronic = 6	13
*Mizrahi *et al	2021	26	Israel	Stent, clips, sealant	SEMS, OTSC, fibrine glue	14	GOJ = 26; distal = 0	Acute = 11; early = 8; chronic = 6 (1 unknown)	26
*Manos *et al	2021	53	France	Stent, endoscopic suturing	Double-pigtail stent, stricturotomy	51	-	Acute = 19; early = 25; chronic = 9	-
*Lorenzo *et al	2018	100	France	Stent, clips	SEMS, double-pigtail stent, OTSC	86	GOJ = 89; distal = 11	Acute = 44; early = 56; chronic = 0	68
*Orive-Calzada *et al	2014	11	Spain	Stent	SEMS	8	-	-	-
*Juza *et al	2015	5	USA	Stent	SEMS	5	GOJ = 3; distal = 2	Acute = 4; early = 1; chronic = 0	-
*Murino *et al	2015	55	Belgium	Stent	SEMS	42	-	-	-
*Nimeri *et al	2016	14	Arab Emirates	Stent	SEMS	14	GOJ = 13; distal = 1	Acute = 11; early = 1; chronic = 2	-
*Spyropoulos *et al	2012	8	Greece	Stent, sealant	SEMS, fibrine glue	8	GOJ = 8; distal = 0	-	4
*Corona *et al	2013	6	Italy	Stent	SEMS	3	-	-	1
*Vix *et al	2015	7	Taiwan	Stent	SEMS	2	GOJ = 7; distal = 0	-	2
*Tan *et al	2010	8	Australia	Stent	SEMS	4	GOJ = 8; distal = 0	Acute = 5; early = 3; chronic = 0	4
*Nedelcu *et al	2015	19	France	Stent	SEMS, double-pigtail stent	19	-	-	2
*van Wezenbeek *et al	2016	7	Netherlands	Stent, clips	SEMS, OTSC	6	GOJ = 7; distal = 0	-	6
*Smallwood *et al	2016	6	USA	EVT, clips	Endoscopic vacuum therapy, OTSC	6	-	-	0
*Simon *et al	2013	9	France	Stent	SEMS	7	GOJ = 9; distal = 0	Acute = 0; early = 6; chronic = 3	2
*Shnell *et al	2017	8	Israel	Endoscopic suturing	Septotomy	8	GOJ = 8; distal = 0	-	0
*Southwell *et al	2016	21	New Zealand	Stent	SEMS	15	GOJ = 15; distal = 6	Acute = 6; early = 12; chronic = 3	10
*Quezada *et al	2015	19	Chile	Stent	FCSEMS	18	GOJ = 19; distal = 0	Acute = 9; early = 6; chronic = 4	10
*Rebibo *et al	2016	20	France	Stent	SEMS, double-pigtail stent	19	-	Acute = 13; early = 4; chronic = 3	3
*Siddique *et al	2020	20	Kuwait	Stent	SEMS, double-pigtail stent	17	-	Acute = 0; early = 9; chronic = 11	2
*Smith *et al	2019	85	USA	Stent, clips, endoscopic suturing	SEMS, OTSC, endoscopic suturing	62	GOJ = 74; distal = 11	Acute = 13; early = 47; chronic = 25	34
*Tringali *et al	2017	10	Italy	Stent	FCSEMS	8	GOJ = 10; distal = 0	-	2
*Shoar *et al	2017	73	USA	Stent, clips	SEMS, OTSC	63	-	-	10
*Sakran *et al	2013	11	Israel	Stent, clips, sealant	SEMS, OTSC, Fibrine glue	5	-	-	-
*Ward *et al	2021	23	USA	Stent, EVT, clips	SEMS, endoscopic vacuum therapy, OTSC	23	-	-	-

*FCSEMS* fully covered self-expanding esophageal metal stents, *OTSC* over-the-scope clip, *EVT* endoscopic vacuum therapy, *GOJ* GastrOesophageal Junction, *SEPS* self-expandable plastic stent, *TTS* through-the-scope clip

### Quality Assessment and Publication Bias

Quality assessment of the included studies according to the Newcastle–Ottawa scale is reported in Table 2. The funnel plot showed symmetry (Supplementary File 2), confirmed by Egger’s regression test (*p* = 0.151), which indicates the absence of publication bias.

**Table 2:** Quality assessment of the included studies according to the Newcastle–Ottawa scale

*STUDY*		Selection	Sample size		Staple-line leak definition	Adjustment for confounders		Staple-line leak healing definition
*Aburajab *et al		High	High		Low	High		Low
*Alamdari *et al		High	High		Low	High		Low
*Assalia *et al		High	High		Low	High		Low
*Baretta *et al		High	High		Low	High		Low
*Archid *et al		High	High		Low	Low		Low
*Manta *et al		High	High		Low	Low		Low
*Bona *et al		High	High		Low	Low		Low
*Bashah *et al		High	High		Low	High		Low
*Yimcharoen *et al		High	High		Low	High		Low
*Aljahdli *et al		High	High		Low	High		Low
*Almadi *et al		High	High		Low	High		Low
*Boerlage *et al		Low	High		Low	High		Low
*Périssé *et al		High	High		Low	High		Low
*Alazmi *et al		High	High		Low	High		Low
*Al-Kurd *et al		High	High		Low	High		Low
*Billman *et al		Low	High		Low	High		Low
*Archid *et al		High	High		Low	High		Low
*Balagué *et al		High	High		Low	Low		Low
*Caiazzo *et al		High	High		Low	High		Low
*Benosman *et al		High	High		Low	Low		Low
*Balagué *et al		High	High		Low	Low		Low
*Fuentes-Valenzuela *et al		High	High		Low	high		Low
*Garofalo *et al		High	High		Low	Low		Low
*Hany *et al		High	High		Low	Low		Low
*Diaz *et al		High	High		Low	Low		Low
*Foo *et al		High	High		Low	High		Low
*Ferraz *et al		High	High		Low	High		Low
*Casella *et al		Low	High		Low	Low		Low
*Donatelli *et al		High	High		Low	Low		Low
*Guzaiz *et al		Low	High		Low	HIgh		Low
*de Moura *et al		High	High		Low	HIgh		Low
*Fishman *et al		High	High		Low	HIgh		Low
*Donatelli *et al		High	High		Low	Low		Low
*Donatelli *et al		High	High		Low	Low		Low
*Csendes *et al		High	High		Low	High		Low
*Christophorou *et al		High	High		Low	High		Low
*Gonzalez *et al		High	High		Low	Low		Low
*Gonzalez *et al		High	High		Low	Low		Low
*El-Sayes *et al		High	High		Low	High		Low
*Lazzarin *et al		High	High		Low	High		Low
*Mahadev *et al		High	High		Low	High		Low
*Mohammad *et al		High	High		Low	High		Low
*Klimczak *et al		High	High		Low	High		Low
*Krishnan *et al		High	High		Low	High		Low
*Talbot *et al		High	High		Low	High		Low
*Leenders *et al		High	High		Low	High		Low
*Moon *et al		High	High		Low	Low		Low
*Lamb *et al		High	High		Low	High		Low
*Pequignot *et al		High	High		Low	Low		Low
*Mercky *et al		High	High		Low	Low		Low
*Leeds *et al		High	High		Low	High		Low
*Keren *et al		High	High		Low	High		Low
*del Campo *et al		High	High		Low	High		Low
*Olmi *et al		High	High		Low	Low		Low
*Mizrahi *et al		High	High		Low	Low		Low
*Manos *et al		Low	High		Low	High		Low
*Lorenzo *et al		High	High		Low	Low		Low
*Orive-Calzada *et al		High	High		Low	High		Low
*Juza *et al		High	High		Low	High		Low
*Murino *et al		High	High		Low	High		Low
*Nimeri *et al		High	High		Low	High		Low
*Spyropoulos *et al		High	High		Low	High		Low
*Corona *et al		High	High		Low	High		Low
*Vix *et al		High	High		Low	High		Low
*Tan *et al		High	High		Low	Low		Low
*Nedelcu *et al		High	High		Low	High		Low
*van Wezenbeek *et al		High	High		Low	High		Low
*Smallwood *et al		High	High		Low	Low		Low
*Simon *et al		High	High		Low	High		Low
*Shnell *et al		Low	High		Low	High		Low
*Southwell *et al		High	High		Low	High		Low
*Quezada *et al		High	High		Low	High		Low
*Rebibo *et al		High	High		Low	Low		Low
*Siddique *et al		High	High		Low	High		Low
*Smith *et al		High	High		Low	High		Low
*Tringali *et al		High	High		Low	High		Low
*Shoar *et al		High	High		Low	High		Low
*Sakran *et al		Low	High		Low	High		Low
*Ward *et al		High	High		Low	low		Low
						**Risk of bias**		
Selection (representativeness of the sample)		Consecutive patients				Low		
		Non-consecutive patients				High		
		Not described				High		
Sample size		Justified				Low		
		Not justified				High		
Detection (staple-line leak definition)		Described				Low		
		Not described				High		
Adjustment for confounders (outcomes reported according to treatment type or timing of treatment)		Adjusted for confounders				Low		
		No adjustment for confounders				High		
Detection (staple-line leak healing definition)		Described				Low		
		No description				High		

### Success Rate of the Endoscopic Treatments

**Fig. 2:**
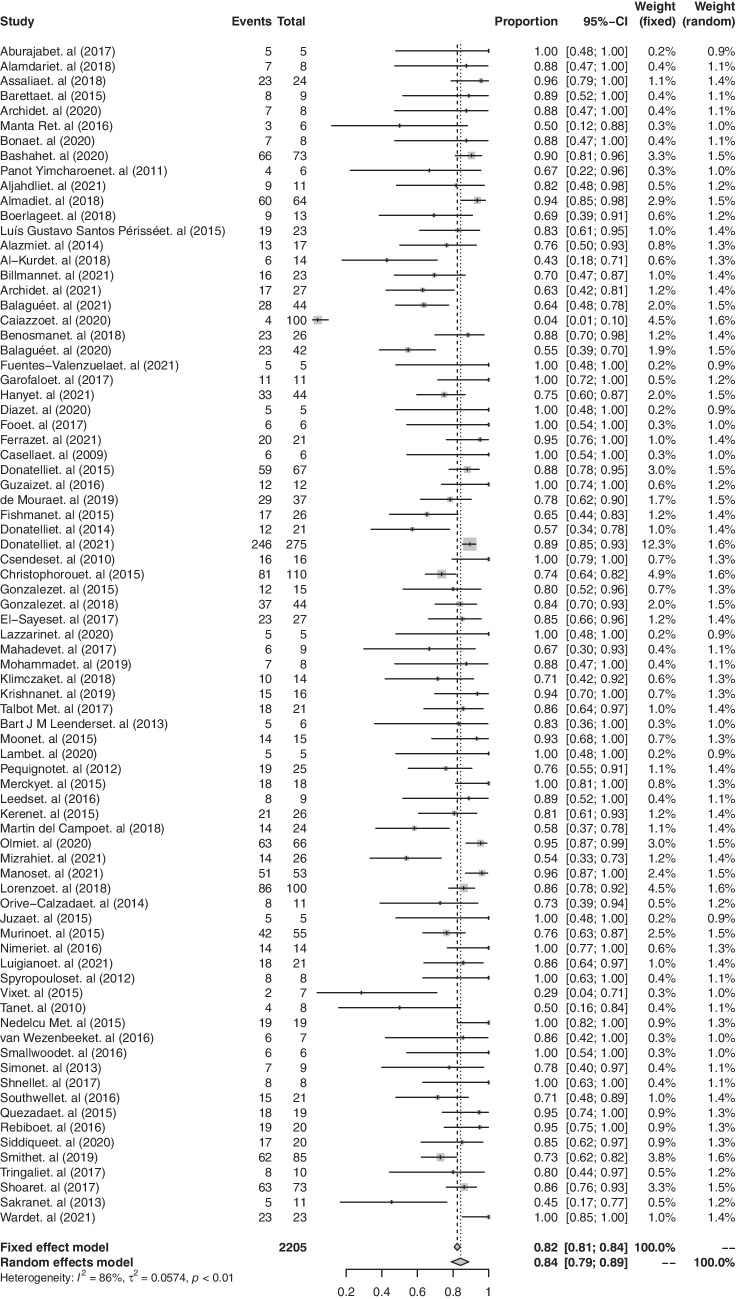
Overall pooled success rate of endoscopic treatments for staple-line leaks

The endoscopic treatment of SLL showed an overall pooled success rate of 84.1% (95% CI, 79.59 to 88.22, Fig. 2) with evidence of high heterogeneity among the studies (*I*^2^ = 81.87%, *Q* = 575.97; *p* < 0.0001). Influence analysis indicated the robustness of this result, showing that, leaving each study out in turn, the summary estimate of the overall success rate varied between − 1.2% and + 0.41%.

Thirteen studies [19, 22, 26, 28, 37, 40, 41, 43, 50, 59, 72–74] reported data about the success rate of endoscopic treatments according to the line of treatment. As first- and second-line treatments, endoscopic methods achieved a success rate of 78.75% (95% CI, 63.78 to 91.07) and 79.12% (95% CI, 63.97 to 91.54), respectively, with no significant difference between the treatments (*p* = 0.172).

Twelve studies [18, 21, 22, 24, 29, 39, 40, 45, 47, 56, 62, 66] reported the treatment success rate according to the timing of treatment. Six studies [22, 37, 41, 43, 50, 59] reported a success rate of 78.1% (95% CI, 48.3 to 98.3) in the case of acute SLL. The success rate in the case of chronic leak was 74.5% (96% CI, 57.8 to 88.7), according to six studies [19, 22, 26, 40, 41, 43]. The success rate in the case of early leak was 84.5% (95% CI, 71.8 to 94.6), according to ten studies [19, 22, 26, 37, 40, 41, 43, 50, 59, 74]. Meta-regression analysis showed that the success rate of endoscopic treatment was independent of the timing of leak occurrence (*p* = 0.543). Also, meta-regression analysis showed that the success rate of the different endoscopic treatment methods was independent of the location of leaks (*p* = 0.053).

After the index endoscopic treatment, 22.62% (95% CI, 16.57 to 29.19) of the patients required an additional procedure according to 63 studies [2, 5, 15–31, 34–47, 50–60, 63–70, 75–86]. A complication related to the endoscopic treatment was reported in 63 studies [7, 15–17, 19–25, 27–38, 40–45, 47, 50–62, 64–71, 75, 77–79, 82–89] with an incidence of 26.7% (95% CI, 21.5 to 32).

### Efficacy of Different Endoscopic Treatments

In total, 70 studies reported the success rates specific to one or more endoscopic treatments.

**Fig. 3:**
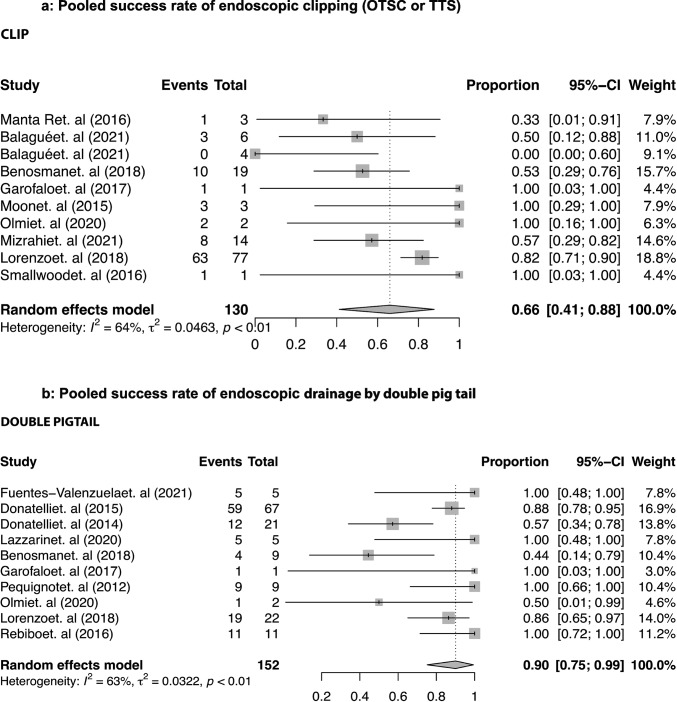
Pooled success rate of the endoscopic clipping (OTSC or TTS) and drainage by double pig tail

**Fig. 4:**
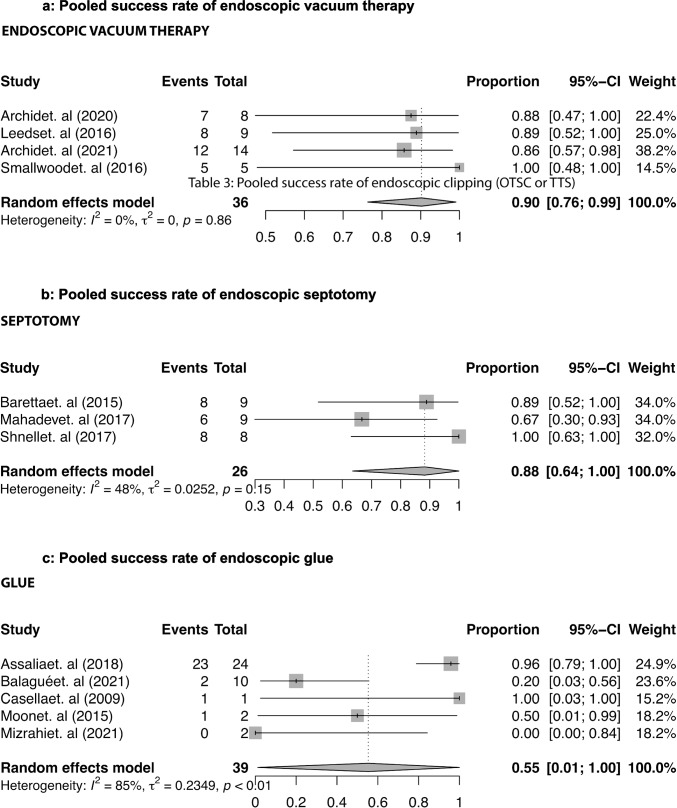
Pooled success rate of the endoscopic vacuum therapy, septotomy, and glue

Ten studies [18, 22, 24, 39, 45, 47, 62, 66, 85, 87] reported a pooled success rate of endoscopic clipping (OTSC or TTS) of 65.98% (95% CI, 41.32 to 87.68) as shown in Fig. 3. Double-pig tail drainage achieved a pooled SLL closure in 90.07% (95% CI, 73.99 to 99.66) of the patients, according to ten studies [23, 24, 27, 34, 40, 45, 47, 56, 62, 64] (Fig. 3). The success rate of endoscopic vacuum therapy was 90.2% (95% CI, 76.31 to 99.09) in four studies [17, 21, 42, 85] (Fig. 4). Three studies [60, 65, 70] reported data on the efficacy of septotomy, with a pooled efficacy observed in 88.25% (95% CI, 63.56 to 100) of the cases (Fig. 4). Sealant application showed a success in 56% (95% CI, 60.3 to 99.3) according to five studies [16, 22, 29, 39, 66] (Fig. 4).

**Fig. 5:**
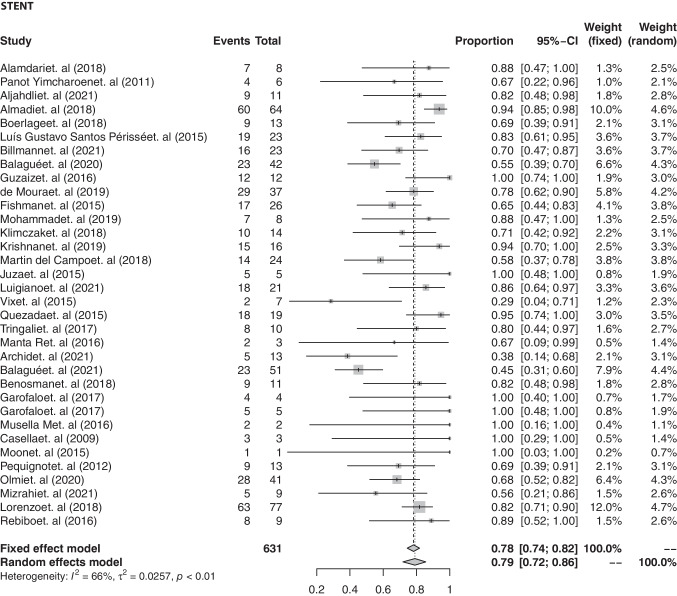
Pooled success rate of the endoscopic stenting

Thirty-three studies [15, 18, 20–22, 24, 29–31, 35, 36, 39, 40, 44, 45, 47, 48, 55–58, 61, 62, 66, 68, 71, 72, 78, 79, 82, 83, 87, 90] reported data on the application of stents, with a pooled success rate of 79.8% (95% CI, 73.4 to 85.64) as shown in Fig. 5.

Meta-regression analysis showed a statistically significant association between the use of double pig tails and a higher success rate (*p* = 0.035). A similar trend was also observed for endoscopic vacuum therapy (*p* = 0.052).

## Discussion

LSG is currently the most commonly performed bariatric procedure worldwide, accounting for up to 75% of the primary interventions in the USA [1, 2]. This popularity is because LSG involves only the stomach, avoiding anastomosis and, consequently, the risk of internal hernias during follow-up. It also preserves the pylorus, allowing normal gastric emptying and a less severe rebound hypoglycemia [1, 91–93]. Another advantage is that, in case of serious complications, it can be converted into a bypass operation.

On the other hand, within the specific complications of LSG, SLL is the most serious, with highly related mortality [6]. The incidence of SLL after primary LSG varies in published series between 0.5 and 7% with a mean value of 2.5%, depending on surgical volume and the surgeon’s experience [4, 7, 94–98]. More recent data show an SLL reduction of over 50%, from 2.5% to approximately 1.1% [4, 99]. However, these figures derived from the registers of the International Societies of Bariatric Surgery are likely underestimated. The ASMBS, evaluating several series of different experienced surgeons, reported a leak rate after LSG between 16 and 20% [4, 96]. Furthermore, the lack of anastomosis and the perceived technical simplicity of the procedure have encouraged many general surgeons to perform LSG in non-specialized bariatric centers. Complications from such cases are often not reported in international registries, leading to significant data loss [6].

The causes of SLL after LSG can be recognized as mechanical or ischemic reasons, both involving increased intraluminal pressure exceeding the strength of the tissue and/or staple line [4, 5]. The clinical and symptomatic appearance of the leak is often subtle; thus, a timely and appropriate treatment is essential to minimize inflammatory and septic complications. However, the patient’s immunoreactive attitude affects more than the therapeutic timing alone in determining the endogenous response. For these reasons, morbidity and mortality can develop despite the correct treatment of the SLL [4, 100].

The endoscopic management of this life-threatening complication has been attempted using several different therapeutic approaches. Unfortunately, the small sample sizes and heterogeneity of published series make direct comparisons unreliable. Many reports refer to patients undergoing multiple, sequential treatments—surgical or endoscopic—making interpretation even more complex [3, 22, 101]. Prospective randomized trials would provide the most reliable evidence, but such studies are unlikely due to practical and ethical limitations. Consequently, no universally accepted treatment algorithm currently exists.

For all these reasons, to address this problem, we conducted a systematic review of the literature and a meta-analysis with meta-regression analysis to identify the best endoscopic treatment of SLL after SG.

Our analysis highlights several key findings. First, we must understand that the endoscopic treatments seem to have similar outcomes as both first- and second-line therapy. This could be because the non-endoscopic first-line therapy (often a radiological drainage) does not modify the anatomic features of the SLL and does not compromise the outcomes of the endoscopic treatments, even if applied as second-line therapies in case of first-line therapy failure.

Second, success rates tend to decline in early, acute, and chronic leaks, although this trend does not reach statistical significance due to the limited number of studies reporting these data (*n* = 6). There is, indeed, a relationship between the timing of the leak and its success treatment rate due to the hardness of the tissue border of the leak: the harder and more callous the edge of the leak, the more difficult it is for endoscopic treatment to be successful [3, 22, 101].

Our findings confirm the superior performance of double-pigtail stents and dedicated mega-stents. This, probably, is because they do not promote direct closure of the leak, but favor healing by secondary intention, together with drainage, for double pig tails, or after radiological drainage, for mega-stents. Perhaps the favorable results of VAC therapy, which stimulates drainage and healing by secondary intention, should be interpreted in this direction.

The worst results, however, are obtained with glues and clips. This depends, mainly, on the dimensions of the leak because the use of biological glues is indicated in case of leaks of a few mm in diameter, anyway less of 1 cm [3, 22, 101], and these situations are typical of very few cases. The evidence on clips is less consistent, as many series pool results for over-the-scope clips (OTSC) and through-the-scope clips (TTSC), despite their differing efficacy. This depends on the dimension and on the timing of the leak, as we already said above. Finally, we must consider that the sleeve is a low-volume and high-pressure system between two functioning sphincters (cardia and pylorus). Thus, even modest increases in endoluminal pressure can exceed the holding force of the clips, leading to a treatment failure [3, 22, 101].

We focused our review on endoscopic modalities because they are recommended as the first-line, conservative approach for SLL; accordingly, we aimed to compare the relative effectiveness of these techniques to inform evidence-based selection within that initial step of care. Surgical management remains integral to a step-up algorithm and is reserved for situations in which non-operative/endoscopic strategies are inappropriate or have failed—namely hemodynamic instability, diffuse peritonitis, or suspected gastric ischemia requiring urgent operative source control; large, devitalized, or otherwise non-closable defects; undrainable or inaccessible collections and ongoing sepsis despite adequate endoscopic and radiologic drainage and refractory leaks after sequential endoscopic attempts, for which several published algorithms advocate early or salvage surgical repair.

## Strengths and Limitations

The strength of the meta-analysis is represented by the high number of studies, which, on the one hand, encompasses all the endoscopic treatments described in the literature and, on the other hand, provides high statistical power, particularly for the meta-regression analyses.

The main limitation is represented by the fact that the vast majority of the included studies were retrospective, with intrinsic methodological limitations, and the observed success rates were derived from highly heterogeneous populations and treatment contexts. Such variability in study design, patient selection, and reporting standards inevitably affects the robustness of pooled estimates. Although statistical heterogeneity was systematically assessed and addressed through subgroup analyses, random-effects modeling, and meta-regression, residual heterogeneity cannot be fully eliminated and should be taken into account when interpreting the findings. Therefore, while our results highlight promising trends, they should not be regarded as conclusive evidence of interchangeability among techniques, but rather as an informed synthesis of the best available—yet imperfect—data. These constraints underscore the need for more rigorous future research. In a setting in which randomized trials are logistically and ethically difficult to perform, prospective multicenter registries with standardized definitions of outcomes, uniform leak classification, and clearly reported endpoints would provide more reliable evidence. Such efforts are essential to translate current evidence into reproducible algorithms that can be consistently applied in clinical practice.

## Supplementary Information

Below is the link to the electronic supplementary material.

ESM 1 MEDLINE search strategy (PNG 32.9 KB)

High Resolution Image (TIF 755 KB)

ESM 2 Supplementary Material 2: Funnel plot showing symmetry, confirmed by Egger’s regression test (*p* = 0.151), which indicates the absence of publication bias (JPEG 26.6 KB) 

## Data Availability

No datasets were generated or analysed during the current study.

## References

[1] Palermo M, Serra E. Laparoscopic sleeve gastrectomy: how do i do it. J Laparoendosc Adv Surg Tech A. 2020;30(1):2–5. 10.1089/lap.2019.0452 31364907

[2] Caiazzo R, Marciniak C, Wallach N, et al. Malignant leakage after sleeve gastrectomy: endoscopic and surgical approach. Obes Surg. 2020;30(11):4459–66. 10.1007/s11695-020-04818-4 32623688

[3] Aurora AR, Khaitan L, Saber AA. Sleeve gastrectomy and the risk of leak: a systematic analysis of 4,888 patients. Surg Endosc. 2012;26(6):1509–15. 10.1007/s00464-011-2085-3 22179470

[4] Kim J, Azagury D, Eisenberg D, et al. ASMBS position statement on prevention, detection, and treatment of gastrointestinal leak after gastric bypass and sleeve gastrectomy, including the roles of imaging, surgical exploration, and nonoperative management. Surg Obes Relat Dis. 2015;11(4):739–48. 10.1016/j.soard.2015.05.001 26071849

[5] Galloro G, Magno L, Musella M, et al. A novel dedicated endoscopic stent for staple-line leaks after laparoscopic sleeve gastrectomy: a case series. Surg Obes Relat Dis. 2014;10(4):607–11. 10.1016/j.soard.2014.02.027 24935179

[6] Galloro G, Ruggiero S, Russo T, et al. Staple-line leak after sleve gastrectomy in obese patients: a hot topic in bariatric surgery. World J Gastrointest Endosc. 2015;7(9):843–6. 10.4253/wjge.v7.i9.843 PMC4515418 26240685

[7] Csendes A, Braghetto I, Leon P, et al. Management of leaks after laparoscopic sleeve gastrectomy in patients with obesity. J Gastrointest Surg. 2010;14(9):1343–8. 10.1007/s11605-010-1249-0 20567930

[8] Stroup DF, Berlin JA, Morton SC, et al. Meta-analysis of observational studies in epidemiology: a proposal for reporting. Meta-analysis of observational studies in epidemiology (MOOSE) group. JAMA. 2000;283(15):2008–12. 10.1001/jama.283.15.2008 10789670

[9] Stang A. Critical evaluation of the Newcastle-Ottawa scale for the assessment of the quality of nonrandomized studies in meta-analyses. Eur J Epidemiol. 2010;25(9):603–5. 10.1007/s10654-010-9491-z 20652370

[10] DerSimonian R, Laird N. Meta-analysis in clinical trials. Control Clin Trials. 1986;7(3):177–88. 10.1016/0197-2456(86)90046-2 3802833

[11] Chen Y, Chen D, Wang Y. Using Freeman-Tukey double arcsine transformation in meta-analysis of single proportions. Aesthetic Plast Surg. 2023;47(Suppl 1):83–4. 10.1007/s00266-022-02977-6 35764810

[12] Biggerstaff BJ, Jackson D. The exact distribution of Cochran’s heterogeneity statistic in one-way random effects meta-analysis. Stat Med. 2008;27(29):6093–110. 10.1002/sim.3428 18781561

[13] Berkey CS, Hoaglin DC, Mosteller F, Colditz GA. A random-effects regression model for meta-analysis. Stat Med. 1995;14(4):395–411. 10.1002/sim.4780140406 7746979

[14] Bowden J, Davey Smith G, Burgess S. Mendelian randomization with invalid instruments: effect estimation and bias detection through Egger regression. Int J Epidemiol. 2015;44(2):512–25. 10.1093/ije/dyv080 PMC4469799 26050253

[15] Malekpour Alamdari N, Shafiee A, Abbasi M, Besharat S. Prevalence and management of gastric leakage after laparoscopic sleeve gastrectomy: a case study in Modarres Hospital, Tehran, Iran. International Journal of Medical Toxicology and Forensic Medicine. 2018 04/01;8(2(Spring)):65–70.

[16] Assalia A, Ilivitzki A, Ofer A, et al. Management of gastric fistula complicating laparoscopic sleeve gastrectomy with biological glue in a combined percutaneous and endoscopic approach. Surg Obes Relat Dis. 2018;14(8):1093–8. 10.1016/j.soard.2018.04.009 29895427

[17] Archid R, Wichmann D, Klingert W, et al. Endoscopic vacuum therapy for staple line leaks after sleeve gastrectomy. Obes Surg. 2020;30(4):1310–5. 10.1007/s11695-019-04269-6 31792702

[18] Manta R, Caruso A, Cellini C, et al. Endoscopic management of patients with post-surgical leaks involving the gastrointestinal tract: a large case series. United European Gastroenterol J. 2016;4(6):770–7. 10.1177/2050640615626051 PMC5386225 28408994

[19] Bona D, Giovannelli A, Micheletto G, et al. Treatment of persistent leaks after laparoscopic sleeve gastrectomy with the simultaneous over-the-scope clip (OTSC) and mega stent strategy. Obes Surg. 2020;30(9):3615–9. 10.1007/s11695-020-04590-5 32291705

[20] Yimcharoen P, Heneghan HM, Tariq N, et al. Endoscopic stent management of leaks and anastomotic strictures after foregut surgery. Surg Obes Relat Dis. 2011;7(5):628–36. 10.1016/j.soard.2011.03.017 21798816

[21] Archid R, Bazerbachi F, Abu Dayyeh BK, et al. Endoscopic negative pressure therapy (ENPT) is superior to stent therapy for staple line leak after sleeve gastrectomy: a single-center cohort study. Obes Surg. 2021;31(6):2511–9. 10.1007/s11695-021-05287-z PMC8113301 33650088

[22] Balague C, Ruiz de Adana JC, Ibarzabal A, et al. Insights into the treatment of postsleeve gastrectomy leak: analysis of the results of 105 cases on a national register. Surg Obes Relat Dis. 2021;17(1):36–43. 10.1016/j.soard.2020.09.001 33097450

[23] Fuentes-Valenzuela E, Garcia-Alonso FJ, Tejedor-Tejada J, et al. Endoscopic internal drainage using transmural double-pigtail stents in leaks following upper gastrointestinal tract surgery. Rev Esp Enferm Dig. 2021;113(10):698–703. 10.17235/reed.2020.7514/2020 33371700

[24] Garofalo F, Noreau-Nguyen M, Denis R, et al. Evolution of endoscopic treatment of sleeve gastrectomy leaks: from partially covered to long, fully covered stents. Surg Obes Relat Dis. 2017;13(6):925–32. 10.1016/j.soard.2016.12.019 28237561

[25] Hany M, Ibrahim M, Zidan A, et al. Role of Primary use of mega stents alone and combined with other endoscopic procedures for early leak and stenosis after bariatric surgery, single-institution experience. Obes Surg. 2021;31(5):2050–61. 10.1007/s11695-020-05211-x 33409972

[26] Diaz R, Welsh LK, Perez JE, et al. Endoscopic septotomy as a treatment for leaks after sleeve gastrectomy: meeting presentations: Digestive Disease Week 2019. Endosc Int Open. 2020;8(1):E70-E5. 10.1055/a-1027-6888 PMC6949161 31921987

[27] Donatelli G, Dumont JL, Cereatti F, et al. Treatment of leaks following sleeve gastrectomy by endoscopic internal drainage (EID). Obes Surg. 2015;25(7):1293–301. 10.1007/s11695-015-1675-x 25913755

[28] Ferraz AAB, Feitosa PHF, Santa-Cruz F, et al. Gastric fistula after sleeve gastrectomy: clinical features and treatment options. Obes Surg. 2021;31(3):1196–203. 10.1007/s11695-020-05115-w 33222105

[29] Casella G, Soricelli E, Rizzello M, et al. Nonsurgical treatment of staple line leaks after laparoscopic sleeve gastrectomy. Obes Surg. 2009;19(7):821–6. 10.1007/s11695-009-9840-8 19381737

[30] de Moura DTH, de Moura EGH, Neto MG, et al. Outcomes of a novel bariatric stent in the management of sleeve gastrectomy leaks: a multicenter study. Surg Obes Relat Dis. 2019;15(8):1241–51. 10.1016/j.soard.2019.05.022 31262650

[31] Fishman S, Shnell M, Gluck N, et al. Use of sleeve-customized self-expandable metal stents for the treatment of staple-line leakage after laparoscopic sleeve gastrectomy. Gastrointest Endosc. 2015;81(5):1291–4. 10.1016/j.gie.2014.11.012 25733128

[32] Christophorou D, Valats JC, Funakoshi N, et al. Endoscopic treatment of fistula after sleeve gastrectomy: results of a multicenter retrospective study. Endoscopy. 2015 Nov;47(11):988–96. 10.1055/s-0034-1392262 26111361

[33] Gonzalez JM, Lorenzo D, Guilbaud T, et al. Internal endoscopic drainage as first line or second line treatment in case of postsleeve gastrectomy fistulas. Endosc Int Open. 2018;6(6):E745-E50. 10.1055/s-0044-101450 PMC5988543 29876512

[34] Lazzarin G, Di Furia M, Romano L, et al. Endoscopic double-pigtail catheter (EDPC) internal drainage as first-line treatment of gastric leak: a case series during laparoscopic sleeve gastrectomy learning curve for morbid obesity. Minim Invasive Surg. 2020;2020:8250904. 10.1155/2020/8250904 PMC7775182 33425388

[35] Mohammad H, Amin MF, Mohamed H, et al. Fully covered metallic mega stents use in the management of post-laparoscopic sleeve gastrectomy leakage, is it beneficial? Surg Chron. 2019;24(3):136–9.

[36] Klimczak T, Klimczak J, Szewczyk T, et al. Endoscopic treatment of leaks after laparoscopic sleeve gastrectomy using MEGA esophageal covered stents. Surg Endosc. 2018;32(4):2038–45. 10.1007/s00464-017-5900-7 29052063

[37] Leenders BJ, Stronkhorst A, Smulders FJ, et al. Removable and repositionable covered metal self-expandable stents for leaks after upper gastrointestinal surgery: experiences in a tertiary referral hospital. Surg Endosc. 2013;27(8):2751–9. 10.1007/s00464-013-2802-1 23436082

[38] Lamb LC, Lawlor MK, Tishler DS, et al. Use of an endoscopic suturing platform for the management of staple line dehiscence after laparoscopic sleeve gastrectomy. Obes Surg. 2020;30(3):895–900. 10.1007/s11695-019-04344-y 31865550

[39] Moon RC, Shah N, Teixeira AF, et al. Management of staple line leaks following sleeve gastrectomy. Surg Obes Relat Dis. 2015;11(1):54–9. 10.1016/j.soard.2014.07.005 25547056

[40] Pequignot A, Fuks D, Verhaeghe P, et al. Is there a place for pigtail drains in the management of gastric leaks after laparoscopic sleeve gastrectomy? Obes Surg. 2012;22(5):712–20. 10.1007/s11695-012-0597-0 22328096

[41] Mercky P, Gonzalez JM, Aimore Bonin E, et al. Usefulness of over-the-scope clipping system for closing digestive fistulas. Dig Endosc. 2015;27(1):18–24. 10.1111/den.12295 24720574

[42] Leeds SG, Burdick JS. Management of gastric leaks after sleeve gastrectomy with endoluminal vacuum (E-Vac) therapy. Surg Obes Relat Dis. 2016;12(7):1278–85. 10.1016/j.soard.2016.01.017 27178614

[43] Keren D, Eyal O, Sroka G, et al. Over-the-scope clip (OTSC) system for sleeve gastrectomy leaks. Obes Surg. 2015;25(8):1358–63. 10.1007/s11695-014-1540-3 25511753

[44] Martin Del Campo SE, Mikami DJ, Needleman BJ, et al. Endoscopic stent placement for treatment of sleeve gastrectomy leak: a single institution experience with fully covered stents. Surg Obes Relat Dis. 2018;14(4):453–61. 10.1016/j.soard.2017.12.015 29370996

[45] Olmi S, Cesana G, Rubicondo C, et al. Management of 69 gastric leakages after 4294 consecutive sleeve: the experience of a high volume bariatric center. Obes Surg. 2020;30(8):3084–92. 10.1007/s11695-020-04658-2 32382961

[46] Manos T, Nedelcu M, Nedelcu A, et al. Leak after sleeve gastrectomy: updated algorithm of treatment. Obes Surg. 2021;31(11):4861–7. 10.1007/s11695-021-05656-8 34455540

[47] Lorenzo D, Guilbaud T, Gonzalez JM, et al. Endoscopic treatment of fistulas after sleeve gastrectomy: a comparison of internal drainage versus closure. Gastrointest Endosc. 2018;87(2):429–37. 10.1016/j.gie.2017.07.032 28750839

[48] Juza RM, Haluck RS, Pauli EM, et al. Gastric sleeve leak: a single institution's experience with early combined laparoendoscopic management. Surg Obes Relat Dis. 2015;11(1):60–4. 10.1016/j.soard.2014.06.011 25543312

[49] Nimeri A, Ibrahim M, Maasher A, et al. Management algorithm for leaks following laparoscopic sleeve gastrectomy. Obes Surg. 2016;26(1):21–5. 10.1007/s11695-015-1751-2 26071239

[50] Tan JT, Kariyawasam S, Wijeratne T, et al. Diagnosis and management of gastric leaks after laparoscopic sleeve gastrectomy for morbid obesity. Obes Surg. 2010;20(4):403–9. 10.1007/s11695-009-0020-7 19936855

[51] Simon F, Siciliano I, Gillet A, et al. Gastric leak after laparoscopic sleeve gastrectomy: early covered self-expandable stent reduces healing time. Obes Surg. 2013;23(5):687–92. 10.1007/s11695-012-0861-3 23315096

[52] Southwell T, Lim TH, Ogra R. Endoscopic therapy for treatment of staple line leaks post-laparoscopic sleeve gastrectomy (LSG): experience from a large bariatric surgery centre in New Zealand. Obes Surg. 2016;26(6):1155–62. 10.1007/s11695-015-1931-0 26475027

[53] Siddique I, Alazmi W, Al-Sabah SK. Endoscopic internal drainage by double pigtail stents in the management of laparoscopic sleeve gastrectomy leaks. Surg Obes Relat Dis. 2020;16(7):831–8. 10.1016/j.soard.2020.03.028 32389513

[54] Smith ZL, Park KH, Llano EM, et al. Outcomes of endoscopic treatment of leaks and fistulae after sleeve gastrectomy: results from a large multicenter U.S. cohort. Surg Obes Relat Dis. 2019;15(6):850–5. 10.1016/j.soard.2019.04.009 31122826

[55] Quezada N, Maiz C, Daroch D, et al. Effect of early use of covered self-expandable endoscopic stent on the treatment of postoperative stapler line leaks. Obes Surg. 2015;25(10):1816–21. 10.1007/s11695-015-1622-x 25840555

[56] Rebibo L, Bartoli E, Dhahri A, et al. Persistent gastric fistula after sleeve gastrectomy: an analysis of the time between discovery and reoperation. Surg Obes Relat Dis. 2016;12(1):84–93. 10.1016/j.soard.2015.04.012 26070397

[57] Aljahdli ES, Aldabbagh A, Salah F, et al. Endoscopic management of post-laparoscopic sleeve gastrectomy leakage and stenosis using fully covered stent. Saudi J Med Med Sci. 2021;9(1):45–50. 10.4103/sjmms.sjmms_347_19 PMC7839568 33519343

[58] Almadi MA, Bamihriz F, Alharbi O, et al. Use of Self-expandable metal stents in the treatment of leaks complicating laparoscopic sleeve gastrectomy: a cohort study. Obes Surg. 2018;28(6):1562–70. 10.1007/s11695-017-3054-2 29235015

[59] Alazmi W, Al-Sabah S, Ali DA, et al. Treating sleeve gastrectomy leak with endoscopic stenting: the Kuwaiti experience and review of recent literature. Surg Endosc. 2014;28(12):3425–8. 10.1007/s00464-014-3616-5 24946741

[60] Baretta G, Campos J, Correia S, et al. Bariatric postoperative fistula: a life-saving endoscopic procedure. Surg Endosc. 2015;29(7):1714–20. 10.1007/s00464-014-3869-z 25294547

[61] Perisse LG, Perisse PC, Bernardo JC. Endoscopic treatment of the fistulas after laparoscopic sleeve gastrectomy and Roux-en-Y gastric bypass. Rev Col Bras Cir. 2015;42(3):159–64. 10.1590/0100-69912015003006 26291256

[62] Benosman H, Rahmi G, Perrod G, et al. Endoscopic management of post-bariatric surgery fistula: a tertiary care center experience. Obes Surg. 2018;28(12):3910–5. 10.1007/s11695-018-3432-4 30074143

[63] Foo JW, Balshaw J, Tan MHL, et al. Leaks in fixed-ring banded sleeve gastrectomies: a management approach. Surg Obes Relat Dis. 2017;13(8):1259–64. 10.1016/j.soard.2017.03.031 28545915

[64] Donatelli G, Ferretti S, Vergeau BM, et al. Endoscopic internal drainage with enteral nutrition (EDEN) for treatment of leaks following sleeve gastrectomy. Obes Surg. 2014;24(8):1400–7. 10.1007/s11695-014-1298-7 24898719

[65] Mahadev S, Kumbhari V, Campos JM, et al. Endoscopic septotomy: an effective approach for internal drainage of sleeve gastrectomy-associated collections. Endoscopy. 2017;49(5):504–8. 10.1055/s-0042-122012 28114687

[66] Mizrahi I, Grinbaum R, Elazary R, et al. Staple line leaks following laparoscopic sleeve gastrectomy: low efficacy of the over-the-scope clip. Obes Surg. 2021;31(2):813–9. 10.1007/s11695-020-05036-8 33047293

[67] Spyropoulos C, Argentou MI, Petsas T, et al. Management of gastrointestinal leaks after surgery for clinically severe obesity. Surg Obes Relat Dis. 2012;8(5):609–15. 10.1016/j.soard.2011.04.222 21616725

[68] Vix M, Diana M, Marx L, et al. Management of staple line leaks after sleeve gastrectomy in a consecutive series of 378 patients. Surg Laparosc Endosc Percutan Tech. 2015;25(1):89–93. 10.1097/SLE.0000000000000026 24752161

[69] van Wezenbeek MR, de Milliano MM, Nienhuijs SW, et al. A Specifically designed stent for anastomotic leaks after bariatric surgery: experiences in a tertiary referral hospital. Obes Surg. 2016;26(8):1875–80. 10.1007/s11695-015-2027-6 26699374

[70] Shnell M, Gluck N, Abu-Abeid S, et al. Use of endoscopic septotomy for the treatment of late staple-line leaks after laparoscopic sleeve gastrectomy. Endoscopy. 2017;49(1):59–63. 10.1055/s-0042-117109 27875853

[71] Tringali A, Bove V, Perri V, et al. Endoscopic treatment of post-laparoscopic sleeve gastrectomy leaks using a specifically designed metal stent. Endoscopy. 2017;49(1):64–8. 10.1055/s-0042-117235 27706525

[72] Musella M, Milone M, Bianco P, et al. Acute leaks following laparoscopic sleeve gastrectomy: early surgical repair according to a management algorithm. J Laparoendosc Adv Surg Tech A. 2016;26(2):85–91. 10.1089/lap.2015.0343 26671482

[73] de Aretxabala X, Leon J, Wiedmaier G, et al. Gastric leak after sleeve gastrectomy: analysis of its management. Obes Surg. 2011;21(8):1232–7. 10.1007/s11695-011-0382-5 21416198

[74] Ward MA, Ebrahim A, Clothier JS, et al. Factors that promote successful endoscopic management of laparoscopic sleeve gastrectomy leaks. Surg Endosc. 2021;35(8):4638–43. 10.1007/s00464-020-07890-0 32780233

[75] Aburajab MA, Max JB, Ona MA, et al. Covered esophageal stenting is effective for symptomatic gastric lumen narrowing and related complications following laparoscopic sleeve gastrectomy. Dig Dis Sci. 2017;62(11):3077–83. 10.1007/s10620-017-4701-0 28815402

[76] Al-Kurd A, Grinbaum R, Abubeih A, et al. Not all leaks are created equal: a comparison between leaks after sleeve gastrectomy and Roux-en-Y gastric bypass. Obes Surg. 2018;28(12):3775–82. 10.1007/s11695-018-3409-3 30022425

[77] Bashah M, Khidir N, El-Matbouly M. Management of leak after sleeve gastrectomy: outcomes of 73 cases, treatment algorithm and predictors of resolution. Obes Surg. 2020;30(2):515–20. 10.1007/s11695-019-04203-w 31707571

[78] Billmann F, Pfeiffer A, Sauer P, et al. Endoscopic stent placement can successfully treat gastric leak following laparoscopic sleeve gastrectomy if and only if an esophagoduodenal megastent is used. Obes Surg. 2022;32(1):64–73. 10.1007/s11695-021-05467-x PMC8752538 34731416

[79] Boerlage TCC, Houben GPM, Groenen MJM, et al. A novel fully covered double-bump stent for staple line leaks after bariatric surgery: a retrospective analysis. Surg Endosc. 2018;32(7):3174–80. 10.1007/s00464-018-6034-2 PMC5988771 29344787

[80] Donatelli G, Spota A, Cereatti F, et al. Endoscopic internal drainage for the management of leak, fistula, and collection after sleeve gastrectomy: our experience in 617 consecutive patients. Surg Obes Relat Dis. 2021;17(8):1432–9. 10.1016/j.soard.2021.03.013 33931322

[81] Granata A, Amata M, Ligresti D, et al. Endoscopic management of post-surgical GI wall defects with the overstitch endosuturing system: a single-center experience. Surg Endosc. 2020;34(9):3805–17. 10.1007/s00464-019-07145-7 31583467

[82] Guzaiz N, Arabi M, Khankan A, et al. Gastroesophageal stenting for the management of post sleeve gastrectomy leak. A single institution experience. Saudi Med J. 2016;37(12):1339–43. 10.15537/smj.2016.12.15761 PMC5303772 27874149

[83] Krishnan V, Hutchings K, Godwin A, et al. Long-term outcomes following endoscopic stenting in the management of leaks after foregut and bariatric surgery. Surg Endosc. 2019;33(8):2691–5. 10.1007/s00464-018-06632-7 30701363

[84] Nedelcu M, Manos T, Cotirlet A, et al. Outcome of leaks after sleeve gastrectomy based on a new algorithm adressing leak size and gastric stenosis. Obes Surg. 2015;25(3):559–63. 10.1007/s11695-014-1561-y 25589019

[85] Smallwood NR, Fleshman JW, Leeds SG, et al. The use of endoluminal vacuum (E-Vac) therapy in the management of upper gastrointestinal leaks and perforations. Surg Endosc. 2016;30(6):2473–80. 10.1007/s00464-015-4501-6 26423414

[86] Talbot M, Yee G, Saxena P. Endoscopic modalities for upper gastrointestinal leaks, fistulae and perforations. ANZ J Surg. 2017;87(3):171–6. 10.1111/ans.13355 PMC5347918 26525773

[87] Balague C, Fernandez-Ananin S, et al. The role of endoprostheses in the treatment of leaks after laparoscopic sleeve gastrectomy. Analysis of a Spanish registry. Cir Esp (Engl Ed). 2020;98(7):373–80. Papel de las endoprotesis en el tratamiento de las fistulas posgastrectomia vertical laparoscopica. Analisis de un registro nacional. 10.1016/j.ciresp.2020.02.011 32600648

[88] Corona M, Zini C, Allegritti M, et al. Minimally invasive treatment of gastric leak after sleeve gastrectomy. Radiol Med. 2013;118(6):962–70. 10.1007/s11547-013-0938-7 23801386

[89] Shoar S, Poliakin L, Khorgami Z, et al. Efficacy and safety of the over-the-scope clip (OTSC) system in the management of leak and fistula after laparoscopic sleeve gastrectomy: a systematic review. Obes Surg. 2017;27(9):2410–8. 10.1007/s11695-017-2651-4 28353180

[90] Luigiano C, Di Leo M, Eusebi LH, et al. Management of leaks following laparoscopic sleeve gastrectomy using specifically designed large covered metal stents. Rev Recent Clin Trials. 2021;16(3):303–8. 10.2174/1574887116666210204142417 33563171

[91] Benaiges D, Goday A, Ramon JM, et al. Laparoscopic sleeve gastrectomy and laparoscopic gastric bypass are equally effective for reduction of cardiovascular risk in severely obese patients at one year of follow-up. Surg Obes Relat Dis. 2011;7(5):575–80. 10.1016/j.soard.2011.03.002 21546321

[92] Leyba JL, Aulestia SN, Llopis SN. Laparoscopic roux-en-Y gastric bypass versus laparoscopic sleeve gastrectomy for the treatment of morbid obesity. A prospective study of 117 patients. Obes Surg. 2011;21(2):212–6. 10.1007/s11695-010-0279-8 20835778

[93] Nocca D, Guillaume F, Noel P, et al. Impact of laparoscopic sleeve gastrectomy and laparoscopic gastric bypass on HbA1c blood level and pharmacological treatment of type 2 diabetes mellitus in severe or morbidly obese patients. Results of a multicenter prospective study at 1 year. Obes Surg. 2011;21(6):738–43. 10.1007/s11695-011-0385-2 21468625

[94] Tucker ON, Szomstein S, Rosenthal RJ. Indications for sleeve gastrectomy as a primary procedure for weight loss in the morbidly obese. J Gastrointest Surg. 2008;12(4):662–7. 10.1007/s11605-008-0480-4 18264685

[95] Jurowich C, Thalheimer A, Seyfried F, et al. Gastric leakage after sleeve gastrectomy-clinical presentation and therapeutic options. Langenbecks Arch Surg. 2011;396(7):981–7. 10.1007/s00423-011-0800-0 21556930

[96] Committee ACI. Updated position statement on sleeve gastrectomy as a bariatric procedure. Surg Obes Relat Dis. 2012;8(3):e21–6. 10.1016/j.soard.2012.02.001 22417852

[97] Arias E, Martinez PR, Ka Ming Li V, et al. Mid-term follow-up after sleeve gastrectomy as a final approach for morbid obesity. Obes Surg. 2009;19(5):544–8. 10.1007/s11695-009-9818-6 19280267

[98] Tagaya N, Kasama K, Kikkawa R, et al. Experience with laparoscopic sleeve gastrectomy for morbid versus super morbid obesity. Obes Surg. 2009;19(10):1371–6. 10.1007/s11695-008-9774-6 19067089

[99] Gagner M. Decreased incidence of leaks after sleeve gastrectomy and improved treatments. Surg Obes Relat Dis. 2014;10(4):611–2. 10.1016/j.soard.2014.04.002 25224165

[100] Al-Sabah S, Ladouceur M, Christou N. Anastomotic leaks after bariatric surgery: it is the host response that matters. Surg Obes Relat Dis. 2008;4(2):152–7; discussion 7–8. 10.1016/j.soard.2007.12.010 18294924

[101] Sakran N, Goitein D, Raziel A, et al. Gastric leaks after sleeve gastrectomy: a multicenter experience with 2,834 patients. Surg Endosc. 2013;27(1):240–5. 10.1007/s00464-012-2426-x 22752283

