# Supplementary figures and images for: Endoscopic Treatment of Staple-Line Leaks After Sleeve Gastrectomy in Patients with Obesity: Which One is the Best Option, if Any? A Systematic Review with Meta-Analysis and Meta-regression
Source: Obes Surg. 2025 Oct 20;35(12):5496–514. doi: 10.1007/s11695-025-08294-6 (PMC12722327; doi:10.1007/s11695-025-08294-6)

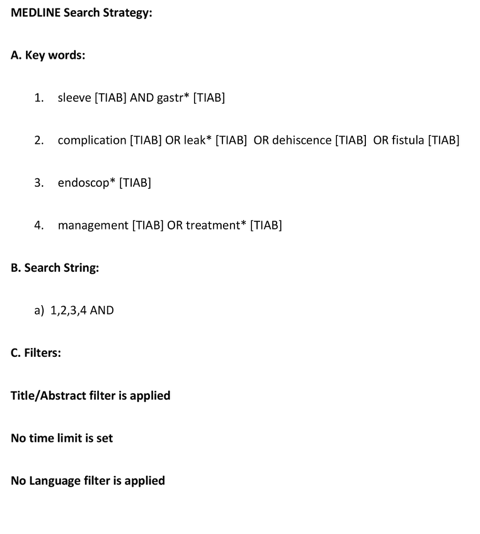

Supplement: Supplementary file 1 — MEDLINE search strategy (PNG 32.9 KB) [file 11695_2025_8294_Fig6_ESM.png]

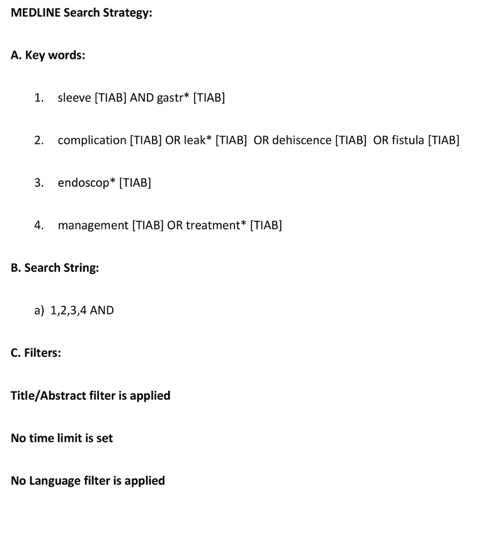

Supplement: Supplementary file 2 — High Resolution Image (TIF 755 KB) [file 11695_2025_8294_MOESM1_ESM.tiff]

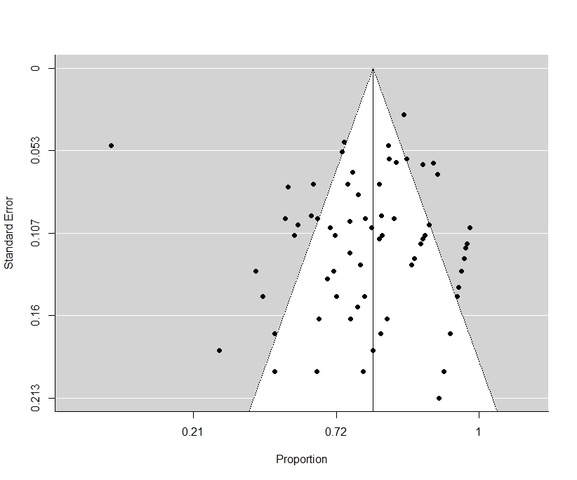

Supplement: Supplementary file 3 — Supplementary Material 2: Funnel plot showing symmetry, confirmed by Egger’s regression test (p = 0.151), which indicates the absence of publication bias (JPEG 26.6 KB) [file 11695_2025_8294_MOESM2_ESM.jpeg]
